# Intraoral Epidermoid Cyst: Case Report and Literature Review

**DOI:** 10.3390/diseases13110358

**Published:** 2025-11-05

**Authors:** Ana Andabak Rogulj, Danica Vidović Juras, Božana Lončar Brzak, Ivana Škrinjar, Bruno Špiljak, Sven Seiwerth

**Affiliations:** 1 Department of Oral Medicine, University of Zagreb School of Dental Medicine, 10000 Zagreb, Croatia; andabak@sfzg.unizg.hr (A.A.R.); djuras@sfzg.unizg.hr (D.V.J.); loncar@sfzg.unizg.hr (B.L.B.); iskrinjar@sfzg.unizg.hr (I.Š.); 2 Clinical Department of Oral Diseases, Dental Clinic, University Hospital Centre (UHC) Zagreb, 10000 Zagreb, Croatia; 3 Department of Pathology, School of Medicine, University of Zagreb, 10000 Zagreb, Croatia; sven.seiwerth@mef.hr

**Keywords:** epidermoid cyst, oral cavity, upper lip, treatment

## Abstract

Background/Objectives: Epidermoid cysts are benign lesions originating from germinative epithelium, characterized by a keratin-filled cavity. They are histopathologically classified as epidermoid, dermoid, or teratoid. Intraoral cases are exceedingly rare, comprising less than 0.01% of all oral cystic lesions, most frequently affecting the floor of the mouth. While usually asymptomatic, they may become painful due to infection or growth. Because of their rarity in sites such as the upper lip, they may be clinically misdiagnosed, making awareness crucial for accurate management. Methods: Diagnosis is primarily clinical and histopathological, with imaging reserved for complex or deep-seated lesions. Complete surgical excision is the standard treatment to prevent recurrence. In this case, diagnostic evaluation included careful clinical inspection, assessment of consistency and mobility, and excisional biopsy with subsequent histopathological confirmation. Results: We report a rare case of a 68-year-old female presenting with a painless, slow-growing swelling of the upper lip. Clinical examination revealed a solitary, whitish, mobile lesion. Histopathological analysis confirmed an epidermoid cyst. The lesion was surgically excised under local anesthesia, with no recurrence observed at six-month follow-up. The outcome highlights the success of surgical management and the importance of monitoring even when the lesion appears benign. Conclusions: Although uncommon in the upper lip, epidermoid cysts should be considered in the differential diagnosis of submucosal swellings. Complete surgical excision offers a favorable outcome and prevents recurrence. Reporting such rare presentations expands clinical awareness and assists in differentiating these lesions from other pathologies of the oral cavity.

## 1. Introduction

Epidermoid (or epidermal) cysts are benign developmental or acquired lesions originating from germinative epithelium. Histologically, they are composed of a cystic cavity lined with stratified squamous epithelium and filled with keratinous material. This keratinous content, typically a thick, yellowish substance, consists of desquamated epithelial cells and keratin—a protein prevalent in skin, hair, and nails. These cysts can appear virtually anywhere on the body but are most frequently encountered in the testicles, ovaries, palms, soles, and scalp [1]. Their ubiquitous distribution reflects the potential of epithelial remnants or traumatic implantation of epithelial cells to give rise to cystic proliferation in almost any anatomical site.

In 1955, Meyer classified these lesions into three histological types: epidermoid, dermoid, and teratoid cysts [2]. Epidermoid cysts consist solely of squamous epithelial lining without skin adnexal structures. Dermoid cysts contain an epidermal lining with skin appendages such as sebaceous glands, sweat glands, or hair follicles [3]. Teratoid cysts are more complex and consist of tissues from all three germ layers, potentially containing muscle, bone, or respiratory/gastrointestinal epithelium [2]. This classification remains clinically important, as the biological behavior, site predilection, and potential for diagnostic confusion vary considerably between the three subtypes.

Intraoral epidermoid cysts are exceedingly rare, accounting for less than 0.01% of all oral cystic lesions [4]. The most frequent site within the oral cavity is the floor of the mouth [5], while cases involving the lips are extremely uncommon [6,7,8,9,10,11,12,13]. Their rarity in these locations often leads to misinterpretation as mucoceles, salivary gland tumors, or other mesenchymal lesions, thereby underscoring the diagnostic challenge they present to oral health professionals. Clinical presentation often includes a painless, slowly enlarging, submucosal nodule that may be firm, soft, or fluctuant depending on the cystic content [14]. These lesions are typically asymptomatic but can become symptomatic due to infection, trauma, or excessive enlargement that interferes with function or aesthetics [1,14]. When occurring in the lips, even small cysts may cause significant esthetic concern, alter lip contour, or interfere with speech and mastication, which amplifies their clinical relevance despite their benign nature.

The diagnostic approach relies on clinical evaluation followed by definitive histopathological analysis of the excised lesion, while advanced imaging techniques such as ultrasonography (US), computed tomography (CT), or magnetic resonance imaging (MRI) may be employed in cases of larger lesions, proximity to vital structures (e.g., salivary ducts, nerves), or suspicion of malignancy [15,16]. Histopathology typically reveals a cystic cavity lined with keratinizing stratified squamous epithelium without adnexal structures, distinguishing epidermoid cysts from dermoid and teratoid subtypes. In selected cases, immunohistochemical markers may also be applied to exclude other keratin-producing lesions. The treatment of choice is complete surgical excision, with emphasis on removing the entire cyst wall to minimize the risk of recurrence [16,17]. Long-term follow-up is advisable, as incomplete removal can result in relapse, although the overall prognosis is excellent.

Here, we present a rare case of an intraoral epidermoid cyst located in the upper lip, alongside a comprehensive review of reported cases involving the lips and buccal mucosa to emphasize the clinical relevance of such lesions. By documenting this case and situating it within the context of existing literature, our aim is to highlight the importance of considering epidermoid cysts in the differential diagnosis of lip swellings, to promote awareness of their clinical and histological hallmarks, and to provide practical insights for their management. Furthermore, this report underlines the need for interdisciplinary collaboration between oral medicine specialists, pathologists, and surgeons, as early recognition and accurate diagnosis are essential to prevent mismanagement. By combining clinical experience with evidence from the literature, we aim to contribute to a better understanding of the epidemiology, diagnosis, and therapeutic outcomes of intraoral epidermoid cysts in rare anatomic locations. The present case is particularly noteworthy as it represents an uncommon presentation of an upper lip epidermoid cyst in an elderly female, in whom the lesion was primarily an esthetic rather than functional concern. The swelling had been long-standing, asymptomatic, and slowly enlarging, without causing impairment in speech or mastication. Such cases emphasize the importance of recognizing inconspicuous but esthetically significant swellings of the lip and confirming the diagnosis through histopathologic evaluation. To the best of our knowledge, this is the first documented case of an intraoral epidermoid cyst of the upper lip occurring in an elderly female without any history of trauma, surgery, or systemic disease. Unlike previously reported cases, most of which involved younger male patients or were associated with congenital or traumatic etiology, this report emphasizes a late-onset, idiopathic presentation. Such presentation broadens the current clinicopathologic spectrum of intraoral epidermoid cysts and underscores the need to consider them even in atypical demographic groups and long-standing, esthetically relevant swellings.

## 2. Case Report

A 68-year-old female was referred to the Department of Oral Medicine, University of Zagreb School of Dental Medicine, with a complaint of a painless, longstanding swelling on the right upper labial mucosa. The lesion had been present for several years without any associated trauma or systemic symptoms. Her medical history included controlled gastroesophageal reflux disease and chronic gastritis, for which she was taking pantoprazole. The patient did not report any history of smoking, alcohol consumption, or previous surgery in the orofacial region, and there was no family history of cystic or neoplastic lesions.

**Figure 1:**
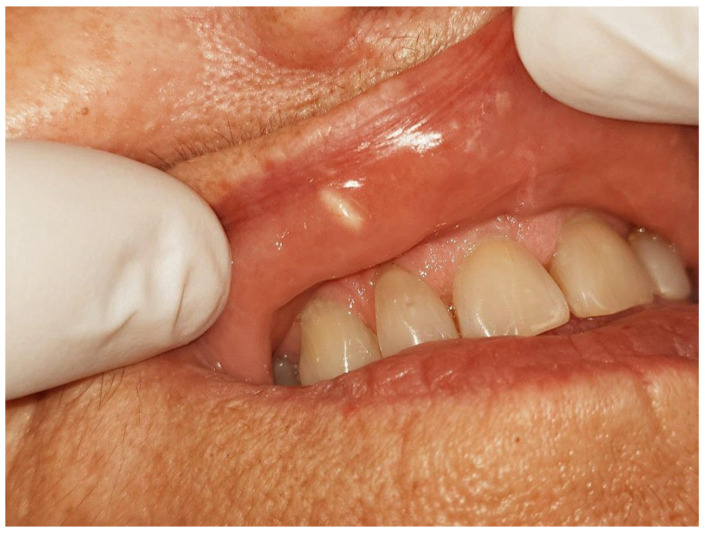
Clinical appearance of the upper lip swelling; a solitary whitish lesion approximately the size of a grain of rice, covered by intact mucosa and asymptomatic on palpation.

Intraoral examination revealed a solitary, well-circumscribed, whitish nodule on the labial mucosa, measuring approximately 0.4 cm in diameter, with a soft and mobile consistency on palpation (Figure 1). The lesion was not associated with tenderness, ulceration, or discharge. Overlying mucosa appeared intact and of normal color, with no secondary changes such as erythema or induration. Regional lymph nodes were not palpable, and extraoral examination showed no facial asymmetry or cutaneous involvement. Based on these features, the initial differential diagnosis included mucocele, minor salivary gland adenoma, fibroma, and lipoma. Given its benign appearance and the patient’s aesthetic concern, an excisional biopsy was planned.

Surgical removal was performed under local anesthesia using an intraoral approach. The lesion was excised in toto with careful preservation of surrounding tissues. Hemostasis was achieved by gentle pressure, and the surgical site was closed with resorbable sutures. No intraoperative complications were encountered, and the procedure was well tolerated.

**Figure 2:**
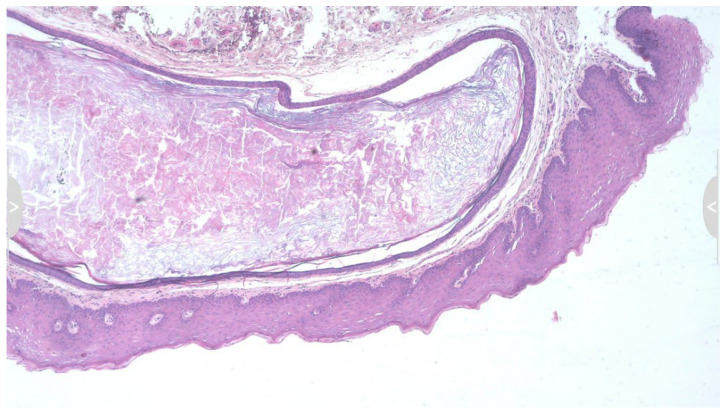
Histopathological examination showing a cystic cavity lined by keratinized stratified squamous epithelium, filled with concentric lamellae of keratin. The cyst wall consists of a thin fibrous capsule without any skin adnexal structures such as sebaceous glands, sweat glands, or hair follicles, thereby confirming the diagnosis of an epidermoid cyst and excluding dermoid cyst (hematoxylin and eosin (HE) stain × 10).

Histopathological examination revealed a cystic cavity lined by stratified squamous epithelium and filled with laminated keratin debris, confirming the diagnosis of an epidermoid cyst (Figure 2). No adnexal structures were observed. The absence of sebaceous or sweat gland components ruled out a dermoid cyst, and the lack of complex tissue derivatives excluded a teratoid cyst. No advanced imaging modalities were employed in this case, as the lesion was small, well-defined, and superficially located in the submucosal tissue of the upper lip. US or MRI was deemed unnecessary due to the absence of deep tissue extension, pain, or rapid growth. The diagnosis was therefore established based on clinical presentation and confirmed by histopathologic examination following surgical excision.

**Figure 3:**
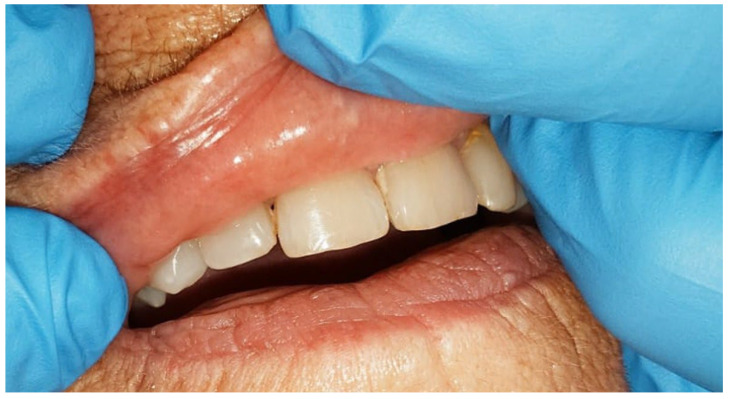
Postoperative outcome six months after excision; the patient remained clinically asymptomatic with completely normal findings and minimal scarring.

Postoperative healing was uneventful, and there was no evidence of recurrence during a six-month follow-up period (Figure 3). At follow-up, the patient reported subjective improvement in lip contour and expressed satisfaction with the aesthetic outcome. Clinical examination revealed complete mucosal healing, with no palpable nodularity or scar hypertrophy, and the site remained functionally and aesthetically stable.

## 3. Literature Research Strategy

To ensure a comprehensive and methodologically transparent overview of intraoral epidermoid cysts affecting the lips and buccal mucosa, a structured literature search was conducted in the PubMed and MEDLINE databases up to 1 June 2025. Both free-text terms and Medical Subject Headings (MeSH) were applied to maximize retrieval sensitivity. The following MeSH terms and their combinations were used: “Cyst, Epidermal,” “Cysts, Epidermal,” “Epidermal Cysts,” “Epidermoid Cyst,” “Cyst, Epidermoid,” “Cysts, Epidermoid,” and “Epidermoid Cysts.” Boolean operators (AND, OR) were used to refine and expand the search queries.

In addition to electronic database searches, the reference lists of relevant studies and prior reviews were manually screened to identify additional eligible reports. Titles, abstracts, and full texts were independently evaluated by two authors (A.A.R. and B.Š.) to confirm that the lesions were located specifically on the lips or buccal mucosa. Predefined inclusion and exclusion criteria were applied to ensure methodological consistency. The inclusion criteria comprised case reports and case series published up to the search date that provided adequate clinical, surgical, and histopathological detail to verify the diagnosis of an epidermoid cyst in the specified anatomic sites. Cases were also required to include basic demographic data, such as patient age, sex, and lesion location, to enable meaningful comparison.

Conversely, reports describing cysts in other intraoral or extraoral regions (e.g., sublingual, submental, submandibular, or cervical areas), as well as studies lacking histopathological confirmation, incomplete case documentation, or insufficient clinical data, were excluded. Reviews, editorials, and duplicate publications were also omitted.

This structured and anatomically focused approach ensured the inclusion of all well-documented cases relevant to the lips and buccal mucosa, providing the most comprehensive synthesis of this subset to date. In contrast to prior reviews predominantly addressing sublingual or submental presentations, the present work offers a targeted consolidation of lip and buccal mucosa cases, thereby enhancing understanding of their clinical spectrum, differential diagnosis, and management considerations.

## 4. Literature Review

Epidermoid cysts are benign, slow-growing lesions that are histologically well-defined and commonly occur in the ovaries, testicles, and skin of the trunk and limbs [1]. Their presence in the oral cavity, however, is an unusual clinical finding. Among intraoral sites, the floor of the mouth is the most frequently involved due to its embryological complexity and proximity to branchial arch fusion sites [5,14]. The predilection for this region has been explained by developmental events during embryogenesis, which facilitate ectodermal entrapment, although sporadic occurrences in other locations demonstrate that traumatic or iatrogenic mechanisms may also play a significant role.

Pathogenesis can be divided into congenital and acquired origins. Congenital cysts are believed to result from ectodermal entrapment during embryogenesis, particularly along fusion planes of the branchial arches [14]. These congenital lesions often manifest during childhood or early adulthood. Acquired epidermoid cysts, on the other hand, may arise following trauma, surgical intervention, or implantation of surface epithelium into deeper tissue planes [14,18]. This distinction, while sometimes theoretical, is important because congenital lesions tend to present earlier and may coexist with other developmental anomalies, whereas acquired forms typically occur later in life and can be linked to identifiable preceding events.

Our review of the literature (Table 1) compiles all reported cases of epidermoid cysts involving the upper lip, lower lip, and buccal mucosa [4,6,7,8,9,10,11,12,13,17,18,19,20,21,22,23,24,25,26,27,28,29,30,31,32,33,34,35,36,37,38]. To our knowledge, only eight cases of epidermoid cysts in the upper lip have been reported, reinforcing the rarity of this lesion in this location [6,7,8,9,10,11,12,13]. These include cases reported by Kuroyanagi, Kawabata and Tooi [6], Moritani et al. [7], Dogan and Bucak [8], Phukan et al. [9], Kim and Hong [10], Mahalakshmi et al. [11], Matsuzaki et al. [12], and Lee, Choi and Kim [13]. This small number highlights the value of every additional case report, which not only adds to the existing body of literature but also contributes to refining diagnostic and therapeutic strategies in oral medicine.

A thorough literature review revealed that these cysts present a wide age distribution, from neonates to the elderly, and are typically characterized by painless, slowly expanding swellings. The clinical features may be vague, and the cysts often go unnoticed unless they reach a size that interferes with function or aesthetics. They usually present as submucosal, non-tender, well-circumscribed nodules with a soft to rubbery consistency. Some lesions may become inflamed or infected, leading to diagnostic confusion with other inflammatory or neoplastic entities [1,14,24,34]. Indeed, their clinical overlap with mucoceles, lipomas, fibromas, and benign salivary gland tumors often necessitates surgical excision for definitive diagnosis.

Consistently, surgical excision was the preferred treatment method, with no reported malignant transformations among these cases. Preoperative imaging, such as US, CT and MRI, was used selectively to determine lesion extent, rule out involvement of deeper structures, and assist with differential diagnosis, particularly when lesions were firm or fixed to underlying tissues [33,39]. Non-invasive imaging methods, particularly US, represent valuable diagnostic tools for superficial cystic lesions of the lips and oral mucosa. US allows real-time, three-dimensional evaluation of lesion size, shape, internal echogenicity, and boundary definition without radiation exposure, facilitating differentiation between cystic, solid, and vascular lesions. Recent studies have emphasized its diagnostic accuracy in assessing oral mucosal and submucosal masses, confirming its utility in preoperative planning and postoperative monitoring [40,41]. This imaging approach delineates the lesion’s exact margins and relationship to adjacent structures, contributing to more precise surgical management and improved prognostic assessment [12,19,27]. While imaging adds value in complex cases, most small, superficial cysts of the lips and buccal mucosa can be safely managed without advanced radiological assessment, thereby reducing patient costs and exposure.

A recurring theme across reported cases is the importance of histopathological confirmation. While imaging may suggest a cystic nature, only microscopic examination can definitively differentiate between an epidermoid cyst and other entities such as dermoid cysts, mucoceles, lipomas, or salivary gland tumors [3,29,30,31]. In all reviewed cases, the cysts were lined by stratified squamous epithelium without adnexal structures, thus confirming their classification as epidermoid. These consistent histological findings reinforce the reliability of histopathology as the gold standard for diagnosis, while also providing reassurance regarding the benign biological behavior of these lesions.

## 5. Discussion

**Figure 4:**
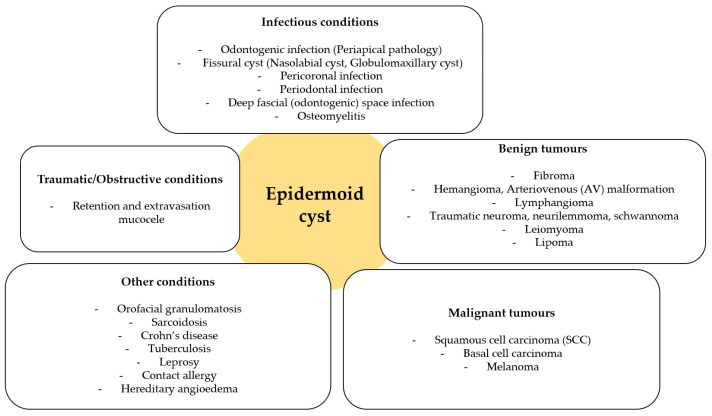
Schematic of differential diagnosis for upper lip masses (an original scheme reproduced from Mahalakshmi et al. [11]).

Although the diagnosis of an epidermoid cyst may appear clinically straightforward, this case represents a distinctly uncommon presentation involving the upper lip mucosa of an elderly female patient, in whom the lesion was asymptomatic and primarily of esthetic concern. Such features, together with detailed surgical and histopathological documentation, highlight the clinical relevance and educational value of this report. The clinical differential diagnosis for upper lip swellings is broad and includes both benign and malignant conditions. Among the most common benign entities are mucoceles, fibromas, lipomas, and benign salivary gland tumors such as canalicular adenomas [11,27,32]. Vascular anomalies and hemangiomas may also be considered, particularly when the lesion is compressible or changes in color [11]. A schematic overview of possible differential diagnoses is provided in Figure 4. In daily practice, the diagnostic process is further complicated by the fact that many of these lesions share overlapping clinical features, and patient history alone is often insufficient to provide a definitive diagnosis. Therefore, careful correlation between clinical, radiological, and histological findings remains the cornerstone of accurate classification. Furthermore, several granulomatous and inflammatory conditions should also be considered in the differential diagnosis of intraoral epidermoid cysts, particularly when the lesion presents as a chronic, painless swelling. These include orofacial granulomatosis, tuberculosis, Crohn’s disease, and sarcoidosis. Orofacial granulomatosis represents a chronic, non-caseating granulomatous disorder of uncertain etiology that may occur idiopathically or in association with systemic diseases such as Crohn’s disease or sarcoidosis. Clinically, it often manifests as localized swelling, cobblestoning, mucosal thickening, or fissuring of the oral mucosa, while histopathological examination typically reveals non-caseating granulomatous inflammation without epithelial lining, which can closely mimic cystic lesions [42,43]. Oral tuberculosis, although rare, may present as ulcerative, nodular, or indurated lesions with granulomatous features and epithelial disruption. Because of its nonspecific clinical appearance, microbiological culture, Ziehl–Neelsen staining, and polymerase chain reaction (PCR) testing are critical for confirming Mycobacterium tuberculosis infection and excluding other causes [44,45]. Crohn’s disease may also involve the oral cavity, producing linear ulcers, diffuse swelling, mucosal tags, or cobblestone-like mucosa that can be mistaken for benign cystic processes. These manifestations may precede intestinal symptoms, emphasizing the importance of oral findings in early diagnosis [46]. Sarcoidosis, a multisystem granulomatous disease, can rarely present with intraoral nodules or swelling of the lips and buccal mucosa, histologically demonstrating compact non-caseating granulomas composed of epithelioid histiocytes and multinucleated giant cells [47,48]. Alongside clinical assessment and medical history, adjunctive laboratory investigations, such as inflammatory markers, tuberculosis screening, Crohn’s-related serologies, serum angiotensin-converting enzyme (ACE) and chitotriosidase (CTO) levels together with chest imaging for sarcoidosis, are often necessary to establish the correct diagnosis and rule out systemic disease [11,43,48]. In contrast to epidermoid cysts, which are well-circumscribed and lined by keratinizing stratified squamous epithelium, granulomatous conditions lack epithelial lining and may involve multiple organ systems. Therefore, comprehensive clinical evaluation, histopathology, and targeted systemic investigations remain essential to distinguish these entities and to guide appropriate multidisciplinary management.

In our case, fibroma and lipoma were initially considered based on the lesion’s superficial location, soft consistency, and non-tender nature. However, the absence of yellowish discoloration made lipoma less likely, while the cystic feel on palpation suggested a possible retention phenomenon or cystic neoplasm. The absence of pulsatility and discoloration also made vascular malformations unlikely. This stepwise exclusion of possibilities illustrates the importance of thorough clinical palpation and inspection, which, even in the absence of advanced imaging, can significantly narrow down the diagnostic spectrum. In addition to fibroma and lipoma, the differential diagnosis of intraoral cystic lesions should also include dermoid and lymphoepithelial cysts. Dermoid cysts share similar clinical characteristics but can be distinguished histologically by the presence of skin adnexal structures such as sebaceous glands, hair follicles, and sweat glands within the cyst wall [49,50]. In contrast, lymphoepithelial cysts are lined by stratified squamous epithelium and are characterized by a subepithelial aggregation of lymphoid tissue containing germinal centers [51,52]. Both lesions typically present as slow-growing, painless swellings in the oral cavity and may clinically mimic epidermoid cysts, underscoring the importance of histopathologic evaluation for accurate diagnosis. When compared with previously published reports, the present case demonstrates distinctive demographic and anatomic characteristics. Most intraoral epidermoid cysts described in the literature have been reported in younger male patients, typically in the second to fourth decade of life, and are more frequently located in the lower lip or buccal mucosa (Table 1). In contrast, our case involved a 68-year-old female with a lesion in the upper lip, representing an uncommon demographic and anatomical presentation. Such variability underscores the importance of recognizing that epidermoid cysts may develop in atypical sites and age groups. The pathogenesis of intraoral epidermoid cysts remains multifactorial, generally classified as either developmental or acquired. Developmental cysts are believed to arise from ectodermal remnants entrapped during the fusion of branchial arches in embryogenesis, whereas acquired (implantation) cysts result from traumatic displacement of epithelial cells into the submucosal tissue following local injury, inflammation, or previous surgical procedures [49,50,51,52].

Although non-invasive imaging such as US or MRI could have been utilized to assess the lesion extent and internal architecture [15,16], the small size and mobile character of the lesion favored a diagnostic and therapeutic excisional biopsy, which remains the standard of care for definitive diagnosis and treatment [17]. In fact, for lesions less than 1 cm in diameter, immediate excision under local anesthesia is often preferred, as it avoids unnecessary diagnostic delays and provides tissue for histopathological confirmation.

Histopathological evaluation is paramount. Epidermoid cysts characteristically exhibit a cystic cavity lined by stratified squamous epithelium and filled with keratinous debris [2,3]. In contrast, as aforementioned, dermoid cysts display skin appendages within the cyst wall, and teratoid cysts may contain elements from all three germ layers [2]. Thus, histological examination not only confirms the diagnosis but also rules out other odontogenic or non-odontogenic cysts, inflammatory pseudocysts, or benign tumors [3]. Moreover, the absence of cellular atypia, mitotic activity, or an invasive growth pattern reassures clinicians of the benign course of these lesions.

Surgical excision is the treatment of choice and is curative in nearly all reported cases. The intraoral approach offers excellent access to labial mucosal lesions, minimizes visible scarring, and allows precise dissection [17]. It is crucial that the cyst be removed intact, as rupture during excision may lead to inflammation, granulomatous reaction, or recurrence [53,54]. Our case followed this protocol, resulting in complete resolution and no signs of recurrence at six months. From a surgical standpoint, early intervention also reduces the likelihood of secondary infection and patient discomfort, while improving cosmetic outcomes.

Although rare, malignant transformation of epidermoid cysts into squamous cell carcinoma has been described in extraoral sites [39]. While this risk appears minimal in the oral cavity, especially in small lesions, complete excision and pathological evaluation remain essential. Additionally, trichoadenoma should be considered in the differential diagnosis, particularly when the lesion presents as a solitary nodule with ambiguous histological features. Trichoadenoma, a rare benign tumor of follicular origin, may mimic epidermoid cysts both clinically and histologically [55]. This overlap further highlights the indispensable role of pathology in guiding final diagnosis and preventing misclassification that could alter patient management.

Notably, the vast majority of oral epidermoid cysts involve the floor of the mouth. The embryologic theory postulates that during the fusion of the first and second branchial arches, ectodermal inclusions can occur, forming a midline mass [5,14]. These developmental anomalies provide a plausible explanation for the predilection of such cysts in the floor of the mouth. However, cases in atypical locations such as the upper lip underscore the need for heightened clinical suspicion and comprehensive evaluation. Indeed, clinicians must remain vigilant, as unusual locations can easily mislead even experienced practitioners, delaying both diagnosis and treatment.

This report documents a rare occurrence of an epidermoid cyst on the upper lip—a location seldom reported in the literature. It emphasizes the necessity of including epidermoid cysts in the differential diagnosis of oral swellings, regardless of location. Despite their rarity, clinical familiarity with such lesions can aid in timely diagnosis and appropriate treatment. Surgical excision remains the gold standard for management, offering both diagnostic clarity and therapeutic resolution. Histopathological analysis is indispensable, not only to confirm the diagnosis but also to rule out malignancy and exclude other cystic or tumor-like lesions. Future studies, particularly larger case series and systematic reviews, are warranted to better define the epidemiological patterns, recurrence risks, and optimal surgical approaches for intraoral epidermoid cysts. Such efforts will enhance evidence-based clinical decision-making and support improved patient care in cases involving these rare but significant lesions. This report adds to the limited body of evidence on lip and buccal mucosa epidermoid cysts, providing detailed clinical, surgical, and histopathological correlation. By consolidating rare but clinically relevant presentations, it enhances awareness among oral medicine specialists and contributes to improved differential diagnosis and patient management.

## 6. Conclusions

Epidermoid cysts of the oral cavity are extremely rare lesions, particularly when located at atypical sites such as the upper lip. Although benign, their clinical presentation often overlaps with more common entities, which may lead to diagnostic uncertainty. Careful clinical assessment combined with histopathological confirmation remains the gold standard for establishing a definitive diagnosis. Complete surgical excision provides both therapeutic resolution and diagnostic clarity, with an excellent prognosis and minimal risk of recurrence when the cyst wall is entirely removed. Documenting such rare cases enriches the available literature, enhances clinical awareness, and supports timely recognition of unusual presentations. Further reports and larger reviews will be essential to broaden our understanding of the pathogenesis, clinical spectrum, and long-term outcomes of intraoral epidermoid cysts. In addition, systematic data collection from multicenter case series could help establish more standardized guidelines for diagnosis and management, particularly in lesions arising at uncommon oral sites. For clinicians, maintaining a high index of suspicion and considering epidermoid cysts in the differential diagnosis of submucosal swellings may prevent misdiagnosis and ensure timely treatment. Ultimately, these efforts will improve patient outcomes and contribute to a more comprehensive understanding of this rare clinical entity.

## Figures and Tables

**Table 1:** Summary of reported epidermoid cyst cases affecting the upper and lower lips and buccal mucosa up to 1 June 2025.

Author and Year (Reference Number)	Sex	Age	Investigation Type of the Published Paper	Onset	History of Trauma	Size (cm) [Laterality]	Symptoms	Diagnostics	Treatment
Upper lip									
Kuroyanagi, Kawabata & Tooi, 1973 [6]	NR	NR	NR	NR	NR	NR	NR	NR	Surgical excision
Moritani et al., 2007 [7]	Male	22	Case report	6 months	Unknown	2.5 × 2.5	Painless mass	MRI, US	Surgical excision
Dogan & Bucak, 2014 [8]	Unknown	3 months	Case report	3 months	No	2.0	Asymptomatic swelling	MRI	Excision biopsy
Phukan et al., 2014 [9]	Male	52	Case report	5 years	No	2.5 × 1.0	Asymptomatic, gradually increasing	Fine-needle aspiration	Excision biopsy
Kim & Hong, 2016 [10]	Male	57	Case report	3 years	Yes	7.0 × 2.0	Slowly growing, erythema, pain	Clinical exam	Excisional biopsy
Mahalakshmi et al., 2016 [11]	Male	38	Case series	3 years	No	3.0 × 2.5	Asymptomatic swelling	Hemogram, ESR	Surgical excision
Matsuzaki et al., 2020 [12]	Male	50	Case report	Longstanding	No	3.0	Painless swelling	Panoramic radiograph, US, MRI	Surgical excision
Lee, Choi & Kim, 2020 [13]	Female	29 months	Case report	2 weeks	No	1.2 × 1.2	Asymptomatic swelling	Periapical radiographs	Excisional biopsy
Andabak Rogulj et al., 2025 (this case)	Female	68	Case report and literature review	Many years	No	0.4	Painless swelling	Clinical exam	Surgical excision
Lower lip									
Ettinger & Manderson, 1973 [19]	Male	59	Case report and literature review	11 years	Yes	0.4 × 0.3	Hard, asymptomatic lump	Panoramic radiograph	Surgical excision
Sewerin & Praetorius, 1974 [20]	Female	86	Case report	Months	No	0.2	Asymptomatic	Clinical exam	Surgical excision
Ishikawa et al., 1976 [38]	Male	42	Case report	NR	Yes	1.8 × 1.0	Painless swelling	Clinical exam	Surgical excision
Papanayotou & Kayavis, 1977 [21]	Male	21	Case report	NR	Yes	NR	NR	NR	Surgical removal
Wang et al., 2005 [22]	Male	13	Case report	1 year	Yes	1.0	Painless swelling	Clinical exam	Excisional biopsy
Shin, Son & Kim, 2007 [23]	Male	31	Case report	10 years	No	3.0 × 3.0	Asymptomatic lump	Punch biopsy	Complete excision
Sable & Jha, 2010 [24]	NR	NR	Case report	NR	NR	NR	Difficulty in speech, esthetics	NR	NR
Amorim et al., 2012 [18]	Male	46	Case report	NR	No	NR	NR	NR	Surgical excision
Kinikar et al., 2016 [25]	Female	27	Case report and literature review	3 years	No	2.0 × 2.0	Asymptomatic swelling	US, FNAC	Surgical excision
Barros et al., 2020 [26]	Male	26	Case report	5 years	Yes	5.0	Painless swelling	Aspiration puncture	Excisional biopsy
Kumar et al., 2023 [27]	Male	29, 23	Retrospective study	1 year, 1 month	Unknown	1.1 × 1.0; 0.5 × 0.5	Painless; mild pain	Clinical exam	Surgical excision
Tharani et al., 2024 [28]	Female	34	Case report	1 month	No	1.0 × 0.5	Painless swelling	Blood tests	Surgical excision
Buccal mucosa									
Schneider & Mesa, 1978 [29]	Female	30, 36	Case reports	3 years, NR	No	3.0, 1.0 [left/right]	NR	NR	Surgical excision
Gutmann et al., 1978 [30]	Female	48	Case report	1 year	Yes	1.5 [right]	Swollen, painful	Clinical exam	Surgical excision
Rajayogeswaran & Eveson, 1989 [31]	Male	25	Case report	1 year	Yes	1.5 × 2.0 [left]	Painful swelling	Clinical exam	Surgical excision
Ozan et al., 2007 [17]	Female	38	Case report	6 months	No	4.0 × 3.0 × 2.0 [left]	Swelling	Clinical exam	Surgical excision
Kini et al., 2013 [32]	Male	25	Case report and review	2 years	No	1.5 × 1.5 × 1.5 [left]	Incidentally noticed	US	Surgical excision
Srivastava et al., 2015 [33]	Male	35	Case report	3 months	No	1.5 × 1.5 [bilateral]	Incidentally noticed	Clinical exam	Excision
Costa et al., 2015 [34]	Male	29	Case report and review	4 years	Yes	3.5 [right]	Painless swelling	Incisional biopsy	Surgical removal
Rohde, Costa & Brinkmeier, 2021 [35]	Female	27 months	Case report	3 weeks	No	4.5 × 3.5 × 1.5 [left]	Asymptomatic	MRI	Excisional biopsy
Dammak et al., 2021 [4]	Male	56	Case report and review	5 years	No	3.4 × 3.1 × 2.1 [left]	Painless swelling	US	Surgical removal
Oh et al., 2023 [36]	Male	47	Retrospective study	Unknown	No	3.0 × 3.0 × 1.5 [right]	Asymptomatic swelling	CT	Enucleation
Kumar et al., 2023 [27]	Male	28, 39, 24, 41	Retrospective study	1–24 months	Unknown	1.2 × 2.0; 1.0 × 0.5; 1.5 × 1.5; 2.0 × 1.5 [bilateral]	Mostly asymptomatic	Clinical exam	Surgical excision
Adhikari et al., 2025 [37]	Male	61, 39	Case series	1.2–2 years	No	3.0 × 2.0 × 2.0; 3.0 × 2.0 × 1.0 [left]	Painless, slowly enlarging	US, FNAC	Surgical excision (intraoral)

Abbreviations: NR = not reported in the manuscript; MRI = magnetic resonance imaging; US = ultrasonography; FNAC = fine-needle aspiration cytology; ESR = erythrocyte sedimentation rate. All sizes are reported to one decimal place, and laterality (left, right, or bilateral) is indicated in parentheses where applicable. Some cases in Table 1 lack specific details due to incomplete data in the original case reports. This limitation reflects the rarity of oral epidermoid cysts and variability in documentation across studies.

## Data Availability

The authors confirm that the data supporting the findings of this study are available within the article.

## Abbreviations

The following abbreviations are used in this manuscript:

CT	Computed Tomography
MRI	Magnetic Resonance Imaging
H&E	Hematoxylin and Eosin
SCC	Squamous Cell Carcinoma
ESR	Erythrocyte Sedimentation Rate
NR	Not Reported in the manuscript
US	Ultrasonography
FNAC	Fine-Needle Aspiration Cytology
ACE	Angiotensin-Converting Enzyme
CTO	Chitotriosidase
